# Reduction of Adhesion Molecule Production and Alteration of eNOS and Endothelin-1 mRNA Expression in Endothelium by *Euphorbia hirta* L. through Its Beneficial β-Amyrin Molecule

**DOI:** 10.3390/molecules190710534

**Published:** 2014-07-18

**Authors:** Mei Fen Shih, Jong Yuh Cherng

**Affiliations:** 1 Department of Pharmacy, Chia-Nan University of Pharmacy & Science, Tainan 717, Taiwan; E-Mail: meifenshih@mail.chna.edu.tw; 2 Department of Chemistry and Biochemistry, National Chung Cheng University, Chia-Yi 621, Taiwan

**Keywords:** E-selectin, sICAM-1, sVCAM-1, Endothelin-1 mRNA, eNOS mRNA, β-amyrin

## Abstract

The inflammatory reaction in large blood vessels involves up-regulation of vascular adhesion molecules such as endothelial cell selectin (E-selectin), soluble vascular cell adhesion molecule (sVCAM)-1, and soluble intercellular adhesion molecule (sICAM)-1. These vascular dysfunctions are associated with the development of atherosclerosis. β-Amyrin, an active component of *Euphorbia hirta* L., has potent anti-inflammatory effects. So far, its preventive effects against the expression of inflammatory mediator-induced adhesion molecules have not been investigated. Endothelial cells (SVEC4-10 cell line) were treated with 50% RAW conditioned media (*i.e.*, normal SVEC4-10 culture media contains 50% of lipopolysaccharide-activated macrophage culture media) without or with β-amyrin (0.6 and 0.3 µM). The production levels of E-selectin, sICAM-1, and sVCAM-1 in the SVEC4-10 cells were measured with ELISA assay kits. Under the same treatment conditions, expression of endothelin (ET)-1 and endothelial type of NO synthase (eNOS) mRNA were analyzed by RT-PCR and agarose gel. With β-amyrin, the 50% RAW conditioned media-induced E-selectin, sICAM-1, and sVCAM-1 levels as well as ET-1 gene expression were all suppressed. β-Amyrin treatment also restored the 50% RAW conditioned media-suppressed eNOS mRNA expression. These data indicate that β-amyrin is potentially useful in preventing chronic inflammation-related vascular diseases.

## 1. Introduction

Inflammation is the pivotal pathologic mechanism of atherosclerosis and contributes to all of its stages, from plaque initiation to growth and rupture [1]. Inflammatory gene expression, increased permeability, leukocyte recruitment and cell turnover have been demonstrated in atherosclerosis-prone sites [2]. Among these, leukocyte recruitment is closely dependent on circulating adhesion molecules (CAMs), which are expressed by the vascular endothelium and believed to play a role in the initiation of the atherosclerotic process. There are several families of CAMs, including integrins, cadherins, selectins and immunoglobulin superfamily members [3]. The most important adhesion molecules involved in atherosclerosis appear to be endothelial cell selectin (E-selectin), intercellular cell adhesion molecule (ICAM)-1, and vascular cell adhesion molecule (VCAM)-1. E-selectin levels in the circulation have been applied to predict cardiovascular disease (CVD) risk in healthy women [4] and to reflect the severity of systemic atherosclerosis [5]. Raised levels of ICAM-1 have been associated with the prediction of cardiovascular events [6,7]. A soluble ICAM-1 (sICAM-1) form of has been found in plasma and identified early stages of inflammation in atherosclerosis [8]. In addition, sICAM-1 levels are elevated in the serum of patients with CVD and several other diseases, and the severity of these diseases has been correlated with serum levels of sICAM-1 [9]. VCAM-1 is thought to be a more specific marker for advanced atherosclerosis, since it is usually expressed in atherosclerotic plaques [10]. In addition, elevated levels of soluble VCAM (sVCAM)-1 are associated with increased risk of coronary events in persons with existing CVD [11,12]. Evidences indicate that lowering circulating levels of adhesion molecules also reduces cardiovascular risk [13,14].

Endothelin-1 (ET-1) is not only a potent vasoconstrictor, but also a chemoattractant for blood monocytes [15]. ET-1 is therefore also implicated in the progression of atherosclerosis [16,17]. Another possible molecule related to atherosclerosis is endothelial type NO synthase (eNOS). eNOS-derived nitric oxide (NO) serves several important functions, including regulation of vascular tone and regional blood flow, suppression of vascular smooth muscle cell proliferation, modulation of leukocyte–endothelial interactions and thrombosis [18]. Further, NO decreases the expression of endothelial surface adhesion molecules, such as VCAM-1 [19].

**Figure 1:**
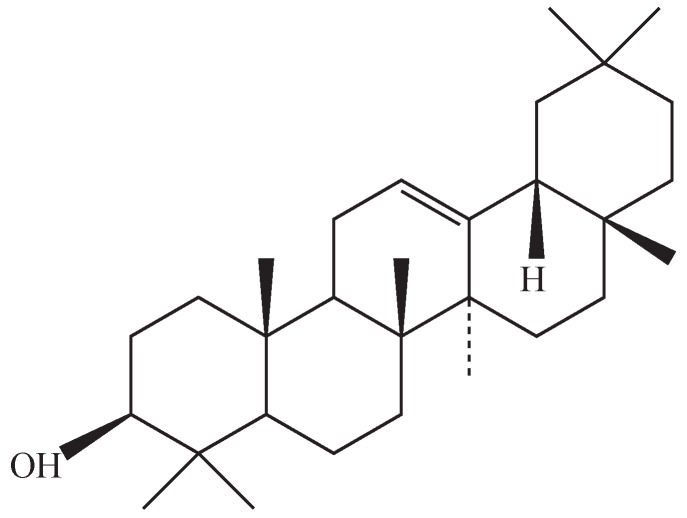
Structure of β-amyrin.

*Euphorbia hirta* L. is used as a folk medicine in Taiwan. Its pharmacological properties include sedative, anxiolytic, analgesic, antipyretic [20], anti-anaphylactic [21], antidiarrheal [22], antibacterial [23,24], antifungal [25], radical scavenging activity [26], diuretic [27] effects, and suppression of the onset of acute allergic phase activities [28]. In addition, the anti-inflammatory effect of *Euphorbia hirta* L., via its active component β-amyrin (Figure 1), has been demonstrated in LPS-activated macrophages [29]. Since atherosclerosis has been recognized as a chronic inflammatory disease, here we investigated the possible preventive effects of β-amyrin on proinflammatory cytokines-mediated adhesion molecule production and expression of ET-1 and eNOS in endothelial cells.

## 2. Results and Discussion

### 2.1. Inhibitory Effects of β-amyrin on Proinflammatory Cytokine-Induced E-selectin, sICAM-1 and sVCAM-1 Production

**Figure 2:**
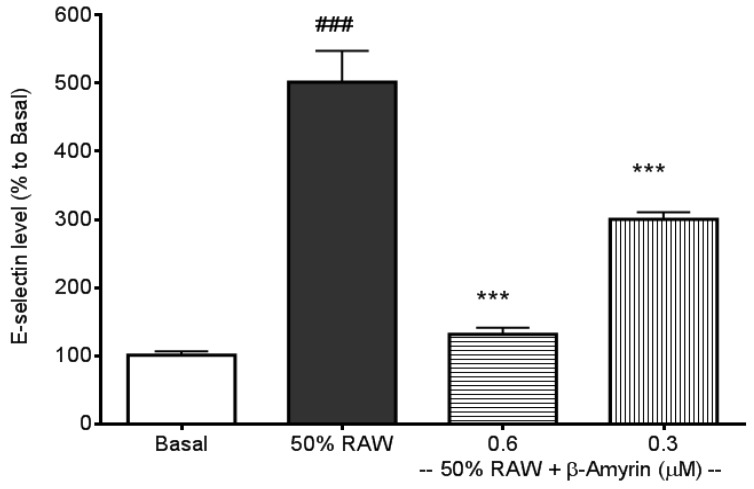
Effects of β-amyrin on proinflammatory cytokine-induced E-selectin production. SVEC4-10 endothelial cells (*n* = 8) were treated with 50% RAW conditioned medium with and without β-amyrin (0.6 and 0.3 µM) for 24 h prior to E-selectin concentration being measured. Statistics are shown for 50% RAW conditioned medium-treated cells. ### *p* < 0.005, compared to the basal; 0.6 and 0.3 µM of β-amyrin. *** *p* < 0.005 compared to 50% RAW conditioned medium-treated group.

**Figure 3:**
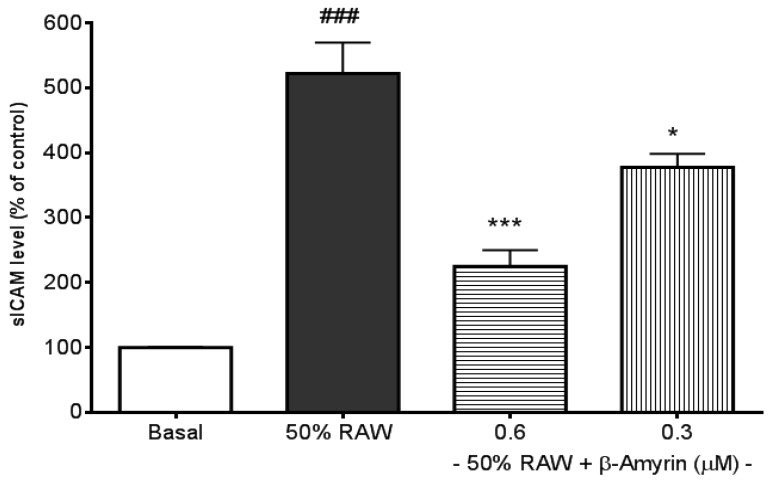
Effects of β-amyrin on proinflammatory cytokine-induced sICAM-1 production. SVEC4-10 endothelial cells (n = 8) were treated with 50% RAW conditioned medium with and without β-amyrin (0.6 and 0.3 µM) for 24 h prior to sICAM-1 concentration being measured. Statistics are shown for 50% RAW conditioned medium-treated cells. ### *p* < 0.005, compared to the basal; 0.6 µM of β-amyrin. *** *p* < 0.005 and 0.3 µM of β-amyrin. * *p* < 0.05 compared to 50% RAW conditioned medium-treated group.

**Figure 4:**
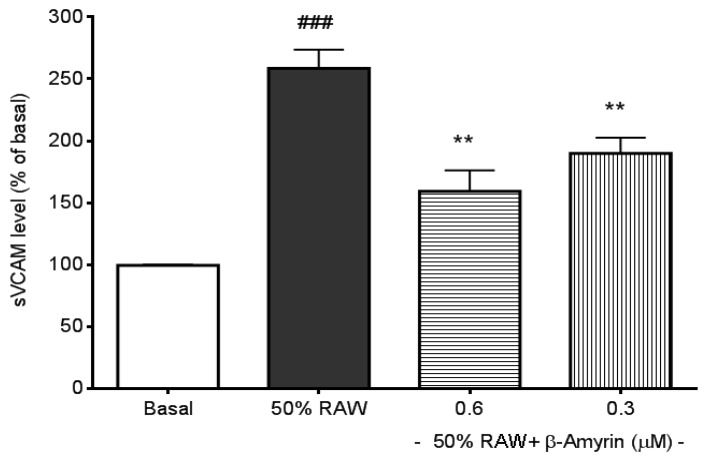
Effects of β-amyrin on proinflammatory cytokine-induced sVCAM-1 production. SVEC4-10 endothelial cells (*n* = 8) were treated with 50% RAW conditioned medium with and without β-amyrin (0.6 and 0.3 µM) for 6 h prior to sVCAM-1 concentration being measured. Statistics are shown for 50% RAW conditioned medium-treated cells ### *p* < 0.005, compared to the basal; 0.6 and 0.3 µM of β-amyrin. ** *p* < 0.01 compared to 50% RAW conditioned media-treated group.

The three most frequently addressed adhesion molecules, E-selectin, sICAM-1, and sVCAM-1, can be induced by several types of proinflammatory cytokines, including TNF-α, IL-1 and IL-6 [30]. LPS‑induced macrophages produce high levels of TNF-α and IL-6 [29,31], therefore, culture media from LPS-activated macrophages were used to stimulate the productions of CAMs. E-selectin production in SVEC4-10 endothelial cells was significantly induced by 50% RAW conditioned medium treatment (*p* < 0.005, Figure 2) compared to normal culture medium-treated endothelial cells (referred to as the basal). The induction was significantly prevented by both 0.6 and 0.3 µM concentrations of β-amyrin (*p* < 0.005). The concentrations of β-amyrin were chosen based on the previous finding [29]. There was about 5-fold increase in sICAM-1 production when SVEC4-10 endothelial cells were cultured with the 50% RAW conditioned medium (*p* < 0.005, Figure 3). The increased sICAM-1 production was significantly inhibited by both 0.6 and 0.3 µM of β-amyrin (*p* < 0.005 and *p* < 0.05, respectively). When SVEC4-10 endothelial cells were treated under the same condition, the production of sVCAM-1 was increased about 2.5 fold to basal (*p* < 0.005, Figure 4). Again, the elevated sVCAM-1 production was also effectively prevented by both concentrations of β-amyrin (*p* < 0.01).

Atherosclerosis is widely accepted to be a chronic inflammatory disease which is initiated by monocyte adhesion to activated endothelial cells [1]. LPS is a powerful bacterial virulence factor to induce inflammatory reactions. Previous studies have shown that LPS induces several major pro‑inflammatory cytokines (including TNF-α, IL-1, IL-6) resulting in vascular inflammation and atherosclerosis [29,30,31]. In this study, we therefore used LPS-activated macrophage culture medium to induce adhesion molecules production in endothelium.

Our results showed that E-selectin and sICAM-1 production were induced by LPS-activated macrophage culture medium into much higher levels than that obtained in sVCAM-1 production. These results were similar to our previous and others’ findings [32,33]. Normally E-selectin is not expressed by endothelial cells. Various cytokines, reactive oxygen species, and bacterial endotoxin can elicit its expression [34]. sICAM-1 is constitutively expressed on endothelial cells in most regional vascular beds, and its expression can be significantly increased by cytokines or bacterial endotoxins. In comparison with sICAM-1, sVCAM-1 predominantly mediates the adhesion of lymphocytes and monocytes upon stimulation [33]. Amberger *et al.* reported a low sVCAM-1 gene expression of human umbilical vein endothelial cells after stimulation of TNF-α [35]. Importantly, all of these elevated adhesion molecules in positive relation to atherosclerosis were substantially reduced by the presence of β-amyrin at both concentrations.

### 2.2. Inhibitory Effects of β-amyrin on Proinflammatory Cytokine-Induced ET-1 Gene Expression

**Figure 5:**
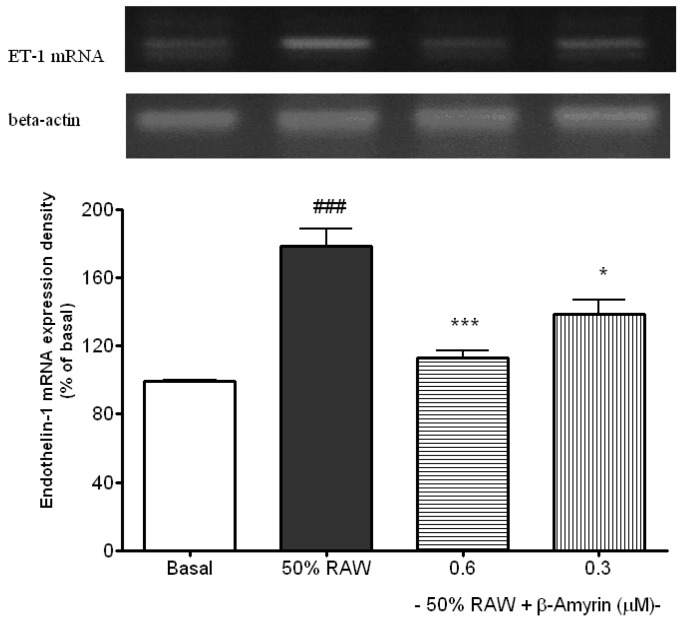
Effects of β-amyrin on proinflammatory cytokine-induced ET-1 mRNA expression. SVEC4-10 endothelial cells (*n* = 8) were treated with 50% RAW conditioned medium with and without β-amyrin (0.6 and 0.3 µM) for 24 h prior to total RNA extraction and PCR were performed. Statistics are shown for 50% RAW conditioned medium-treated cells. ### *p* < 0.005 compared to the basal; 0.6 µM of β-amyrin. *** *p* < 0.005 and 0.3 µM of β-amyrin. * *p* < 0.05 compared to 50% RAW conditioned medium-treated group.

ET-1 mRNA expression was much higher in 50% RAW conditioned medium treated SVEC4-10 endothelial cells compared to the basal (*p* < 0.05, Figure 5). Both 0.6 (*p* < 0.005) and 0.3 (*p* < 0.05) µM of β-amyrin significantly inhibited the induction. ET-1 is synthesized and secreted by vascular endothelial cells. It is not only a potent endogenous vasoconstrictor, but also a potent stimulant of vascular smooth muscle cell proliferation. Therefore, it is believed to play an important role in the development of various circulatory disorders, including hypertension and atherosclerosis. ET-1 mRNA expression was elevated in the artery of atherosclerotic lesion [36] while antagonism of the ET-1 receptors reduced atherosclerotic lesion formation [37]. Thus, blocking ET-1 is considered to be one of the strategies for the prevention of atherosclerosis [17].

### 2.3. Ameliorative Effects of β-amyrin on Proinflammatory Cytokine-Suppressed eNOS mRNA Expression

**Figure 6:**
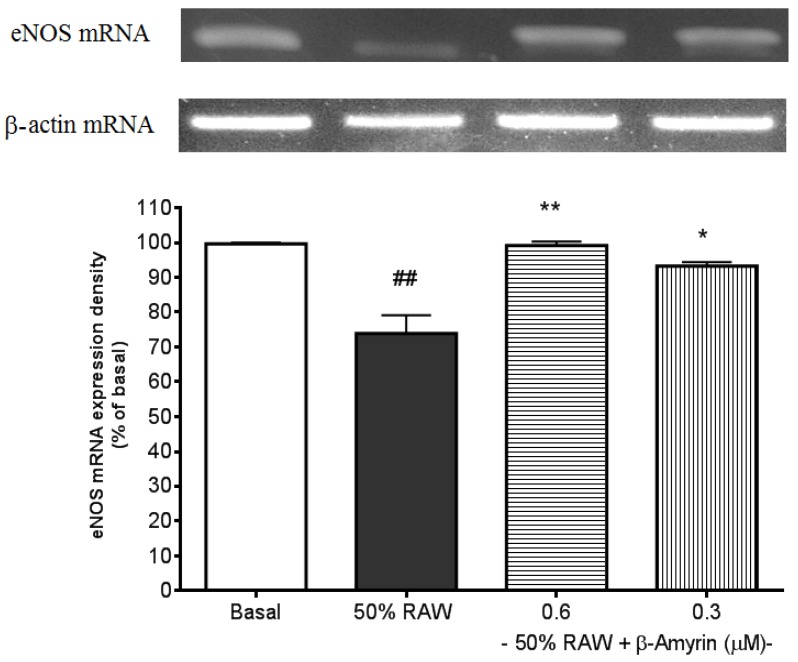
Effects of β-amyrin on proinflammatory cytokine-induced eNOS mRNA expression. SVEC4-10 endothelial cells (*n* = 8) were treated with 50% RAW conditioned medium with and without β-amyrin (0.6 and 0.3 µM) for 24 h prior to total RNA extraction and PCR were performed. Statistics are shown for 50% RAW conditioned medium-treated cells. ## *p* < 0.01 compared to the basal; 0.6 µM of β-amyrin. ** *p* < 0.01 and 0.3 µM of β-amyrin. * *p* < 0.05 compared to 50% RAW conditioned medium-treated group.

Expression of eNOS mRNA in SVEC4-10 endothelial cells was suppressed by 50% RAW conditioned medium treatment (*p* < 0.01, Figure 6). Both 0.6 (*p* < 0.01) and 0.3 (*p* < 0.05) µM of β-amyrin significantly prevented the suppression.

In vessels, NO is constitutively produced from the endothelium by eNOS, which is activated by mechanical stresses such as blood flow-mediated shear stress and stimulated with vasoactive substances such as bradykinin and acetylcholine. In addition, NO controls vascular tone, inhibits monocyte and leukocyte adhesion to the endothelium, inhibits platelet aggregation, decreases endothelial permeability, and inhibits vascular smooth muscle cell migration and proliferation [38]. Recently, eNOS pathway has also been regarded as an anti-atherogenic molecule through its inhibitory effects on adhesion molecules expressions [39]. Many studies have shown that the direct effects of TNF-α on eNOS are via down-regulating eNOS expression [40].

### 2.4. Pathways of β-amyrin’s Effectiveness Leading to Prevent the Development of Atherosclerosis

**Figure 7:**
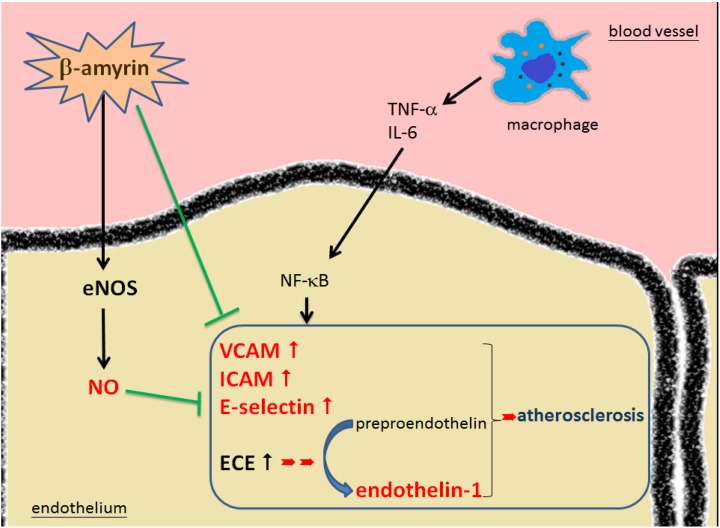
The effectiveness of β-amyrin modulates the development of atherosclerosis via adhesion molecules.

The expression of cellular adhesion molecules (CAMs) by activated endothelial cells under the influence of proinflammatory cytokines is a rate-determining step in the recruitment of inflammatory cells, and the role of endothelial CAMs in CVD has been established [34,41]. In this study, we have found that β-amyrin effectively inhibited the CAMs involved in the development of atherosclerotic initiation induced by proinflammatory cytokines in SVEC4-10 endothelial cells. In addition, β-amyrin successfully suppressed proinflammatory cytokine-induced ET-1 mRNA and normalized eNOS mRNA expression in SVEC4-10 endothelial cells. These beneficial factors make β-amyrin a promising molecule in preventing the development of atherosclerosis (Figure 7).

Recently, these adhesion molecules, such as ICAM and VCAM, were reported to be involved in periodontitis [42], colitis [43], and atopic dermatitis [44]. Since β-amyrin has shown in this study beneficial effects on inflammation through modulation of the adhesion molecules, the use of β-amyrin might be also effective in treatment of adhesion molecule-mediated inflammatory diseases.

## 3. Experimental Section

### 3.1. Materials

Dulbecco’s modified eagles medium (DMEM), sodium pyruvate and non-essential amino acid were purchased from Gibco BRL (Taipei, Taiwan). β-amyrin and lipopolysaccharide (LPS) were purchased from the Extrasynthese (Taipei, Taiwan) and Fluka (Taipei, Taiwan), respectively. A Total RNA Miniprep System (Viogene, Taipei, Taiwan) and Access RT-PCR (Promega, Taipei, Taiwan) were used for RT-PCR. sVCAM-1, sICAM-1 and E-selectin ELISA assay kits were obtained from R&D Systems (Taipei, Taiwan).

### 3.2. Adhesion Molecules — E-selectin, sICAM-1 and sVCAM-1 Level Measurement

SVEC4-10 cells (ATCC number: CRL-2181), which are well differentiated, responding like normal endothelial cells to some interleukins and to extracellular matrix signals for tube-like differentiation. SVEC4-10 was demonstrated to retain morphological and functional characteristics of normal EC [45]. SVEC4-10 endothelial cells were obtained from Bioresource Collection & Research Center (Hsinchu city, Taiwan) and cultured in Dulbecco’s Modified Eagle’s Medium (DMEM) supplemented with 10% Fetal Bovine Serum, 1 mM sodium pyruvate, 100 U/mL penicillin and 100 µg/mL streptomycin. Cells were maintained in 100 mm petri dishes at 37 °C in a humidified atmosphere containing 5% CO_2_. The SVEC4-10 cells were seeded at a density of 2 × 10^4^ cells/well in 96-well plates overnight prior to treatments. For stimuli, the culture medium of SVEC4-10 cells was changed to “50% RAW-conditioned medium” [32] (made of equal volume of SVEC4-10 medium and the supernatant of LPS-stimulated RAW264.7 culture medium) with and without β-amyrin. Cells were incubated for further 24 h prior to E-selectin and sICAM-1 assays and 6 h prior to sVCAM-1 assay [32]. The concentration of these adhesion molecules were measured with commercial ELISA assay kits. RAW 264.7 macrophages were activated by LPS (1 µg/mL) for 24 h prior to the media were collected and frozen at −70 °C. The LPS-stimulated RAW 246.7 cell culture media contained approximately 1.5 ng/mL of TNF-α and 0.35 ng/mL of IL-6 as shown in previous study [29].

### 3.3. RNA Preparation and ET-1 and eNOS RNA Analysis by Reverse Transcription-Polymerase Chain Reaction

SVEC4-10 endothelial cells (1 × 10^6^ cells/well) in 100 mm plates were treated under the same conditions as described above and the incubation time was 24 h. Total RNA was extracted by using a R-5000A RNA isolation kit (Gentra, Minneapolis, MN, USA). The extract of total RNA was reverse transcribed using a first strand cDNA synthesis kit for reverse transcription-polymerase chain reaction (RT-PCR) (Access RT-PCR system, Promega). Semi-quantitative PCR was performed using primers for mouse ET-1 (forward, 5'-AAG CGC TGT TCC TGT TCT TCA-3'; reverse, 5'-CTT GAT GCT ATT GCT GAT GG-3'), eNOS (forward, 5'-GCC CTG TAC CTC AAG ACG CT-3' and reverse, 5'-AAT ACC TGC AGC TTT CCC CA-3'), and housekeeping gene β-actin (forward, 5'-GTG GGC CGC TCA GGC CA-3'; reverse, 5'-CTC AGC TGT GGT GGT GAA GC-3'). Reaction products were electrophoresed in 1.0% agarose gels and visualized with ethidium bromide. Densitometric analysis was performed using the Alpha Imager 2000 Documentation & Analysis System (Alpha Innotech Corporation, San Leandro, CA, USA).

### 3.4. Statistical Analysis

Data from E-selectin, sICAM-1, and sVCAM-1 assays in each group (*n* ≥ 8) were combined from at least two different experimental days. Electrophoresis gel data from each group (*n* ≥ 3) were from at least three different experimental days. A typical one is presented. A two-tailed student’s unpaired *t*-test was used to compare the mean values of two populations of continuous data that were part of a normal distribution. Statistical analyses were based on Student’s *t*-test using Prism software [46].

## 4. Conclusions

β-Amyrin effectively inhibited the cellular molecules involved in the development of atherosclerotic initiation induced by pro-inflammatory cytokines in SVEC4-10 endothelial cells via activation of the eNOS and attenuation of adhesion molecules expressions. Taken together, this evidence suggests β-amyrin is a potentially useful chemical in ameliorating the development of atherosclerosis.
